# Staple aneurysmorrhaphy and suture venoplasty for repair of large bilateral external iliac vein aneurysms in an adolescent

**DOI:** 10.1016/j.jvscit.2022.06.004

**Published:** 2022-07-06

**Authors:** Claire A. Ostertag-Hill, Steven J. Fishman, Heung Bae Kim

**Affiliations:** a Vascular Anomalies Center, Department of Surgery, Boston Children's Hospital, Boston, MA; b Department of Surgery, Boston Children's Hospital, Boston, MA

**Keywords:** Iliac vein, Thrombosis, Venoplasty, Venous aneurysm, Venous malformation

## Abstract

Aneurysms of the iliac veins are very rare; thus, the best approach to management has not yet been defined. We have presented the case of a 17-year-old boy with incidentally identified large bilateral external iliac vein aneurysms. Given the risks of potentially fatal thromboembolism or rupture, he underwent definitive repair of his aneurysms using staple aneurysmorrhaphy combined with additional vein tailoring by suture venoplasty, a technique not previously described for these aneurysms. We have also discussed the etiology, presentation, and our surgical technique to manage this rare condition.

Aneurysms of the iliac veins are exceedingly rare, especially in the absence of an underlying arteriovenous fistula or venous outflow obstruction.^1^^,^^2^ Given the rarity of this condition, the optimal approach to management has not yet been identified.^1^ We have presented the case of an adolescent boy with very large bilateral external iliac veins aneurysms that were repaired using staple aneurysmorrhaphy with additional tailoring by suture venoplasty. The patient provided written informed consent for the report of his case details and imaging studies.

## Case report

A 17-year-old boy was referred to our Vascular Anomalies Center because of an incidental finding of bilateral large external iliac vein aneurysms. He had recently developed an episode of self-resolving hematuria after being hit in the abdomen by a ball, prompting an abdominal imaging study. He denied lower extremity or groin pain, lower extremity swelling, chest pain, and shortness of breath. Additionally, he denied a history of abdominal or pelvic trauma, prior surgical intervention, and a family history of vascular anomalies or connective tissue disorders. The physical examination findings were unremarkable, including no findings to suggest additional venous anomalies or an underlying connective tissue disorder.

**Fig 1:**
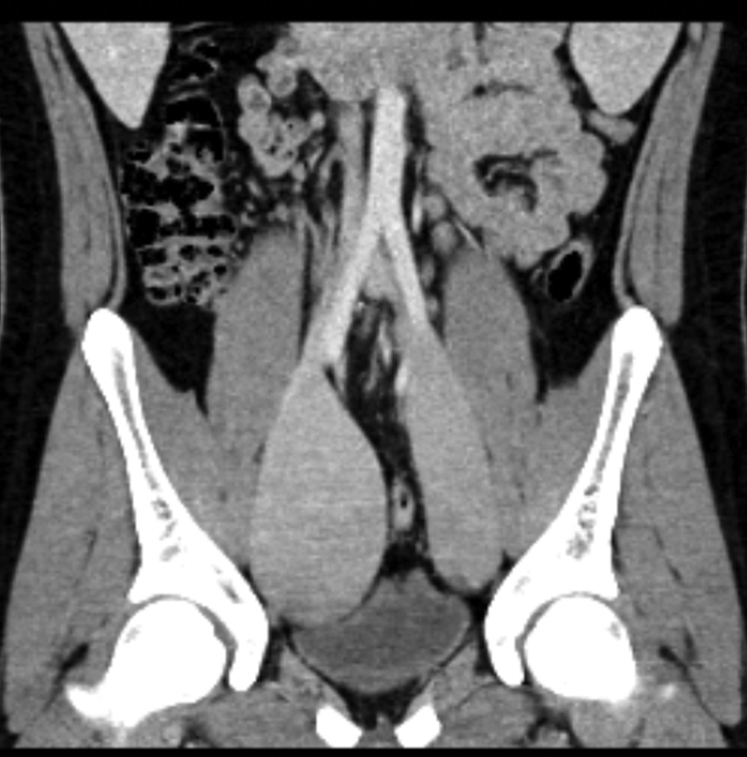
Coronal computed tomography image showing bilateral external iliac vein aneurysms.

Computed tomography angiography confirmed the presence of large bilateral iliac vein aneurysms extending nearly from the inferior vena cava confluence to the internal aspect of the inguinal canal (Fig 1). The right external iliac vein aneurysm measured 6.8 × 6.0 cm, with a length of 14 cm, and the left measured 4.5 × 3.3 cm, with a length of 13 cm. Aneurysmal dilation extended bilaterally to involve the proximal common and distal internal iliac veins. Venous phase imaging suggested the presence of altered flow dynamics. A mild mass effect on the bladder and narrowing of the sigmoid colon were present, with no evidence of venous compression, upstream obstruction, arteriovenous fistula, or thrombosis.

Given the risks of thrombosis, pulmonary embolism, and spontaneous rupture, surgical repair was recommended. After a multidisciplinary discussion, low-molecular-weight heparin was initiated in the interim to decrease the risk of thromboembolism since the history of these aneurysms could not be clearly defined without prior imaging studies.

**Fig 2:**
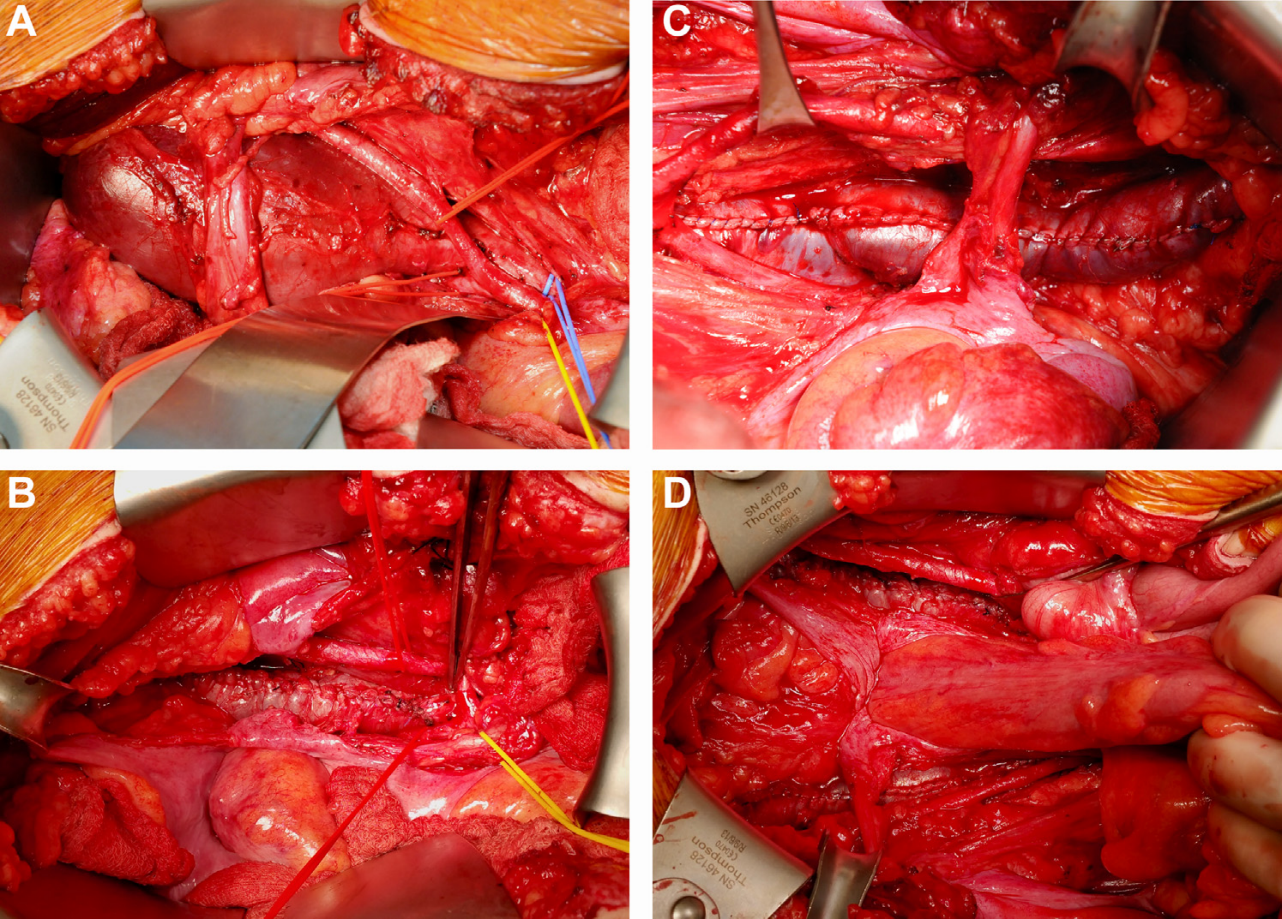
Intraoperative images showing right external iliac artery aneurysm **(A)**, right external iliac artery aneurysm after staple aneurysmorrhaphy and suture venoplasty **(B)**, left external iliac artery aneurysm after staple aneurysmorrhaphy and suture venoplasty **(C)**, and bilateral external iliac artery aneurysms after staple aneurysmorrhaphy and suture venoplasty **(D)**.

The patient was taken to the operating room electively for excision. The bilateral external iliac vein aneurysms were exposed through a lower laparotomy incision (Fig 2, *A*), with care taken to identify and protect the vas deferens, ureter, and gonadal vessels. The aneurysms were carefully separated from the iliac arteries. Vessel loops were placed for proximal and distal control at the level of the junction with the common iliac vein and at the inguinal ligament, respectively. Circumferential dissection of the aneurysms was performed. To repair the aneurysm in our patient, a fully grown adolescent, we elected to perform bilateral staple aneurysmorrhaphy, combined with additional suture venoplasty to tailor the veins.

We started with the larger right-sided aneurysm. First, the aneurysm was manually compressed. Multiple loads of an Endo GIA stapler (Medtronic, Dublin, Ireland) were fired longitudinally along the anterior portion of the vein, using the nonaneurysmal proximal and distal vein as a guide and intentionally leaving the vein larger than normal to allow for additional tailored tapering using suture venoplasty. Running 5-0 Prolene suture was used along the length of the staple line, imbricating the vein further with each bite to tailor it to a size that was intentionally somewhat larger than a normal iliac vein (Fig 2, *B*). At completion, the shape and size match between the proximal and distal nonaneurysmal segments of the vein were excellent, with good flow visualized via Doppler ultrasound examination to ensure the manipulation had not caused thrombosis. The same procedure was then performed on the left aneurysm, with similarly excellent size, shape, and venous flow found at completion (Fig 2, *C* *and* D). Postoperatively, the patient continued receiving a therapeutic heparin infusion until postoperative day 5, when he was transitioned to low-molecular-weight heparin. He was discharged home the next day with a prescription for anticoagulation therapy.

**Fig 3:**
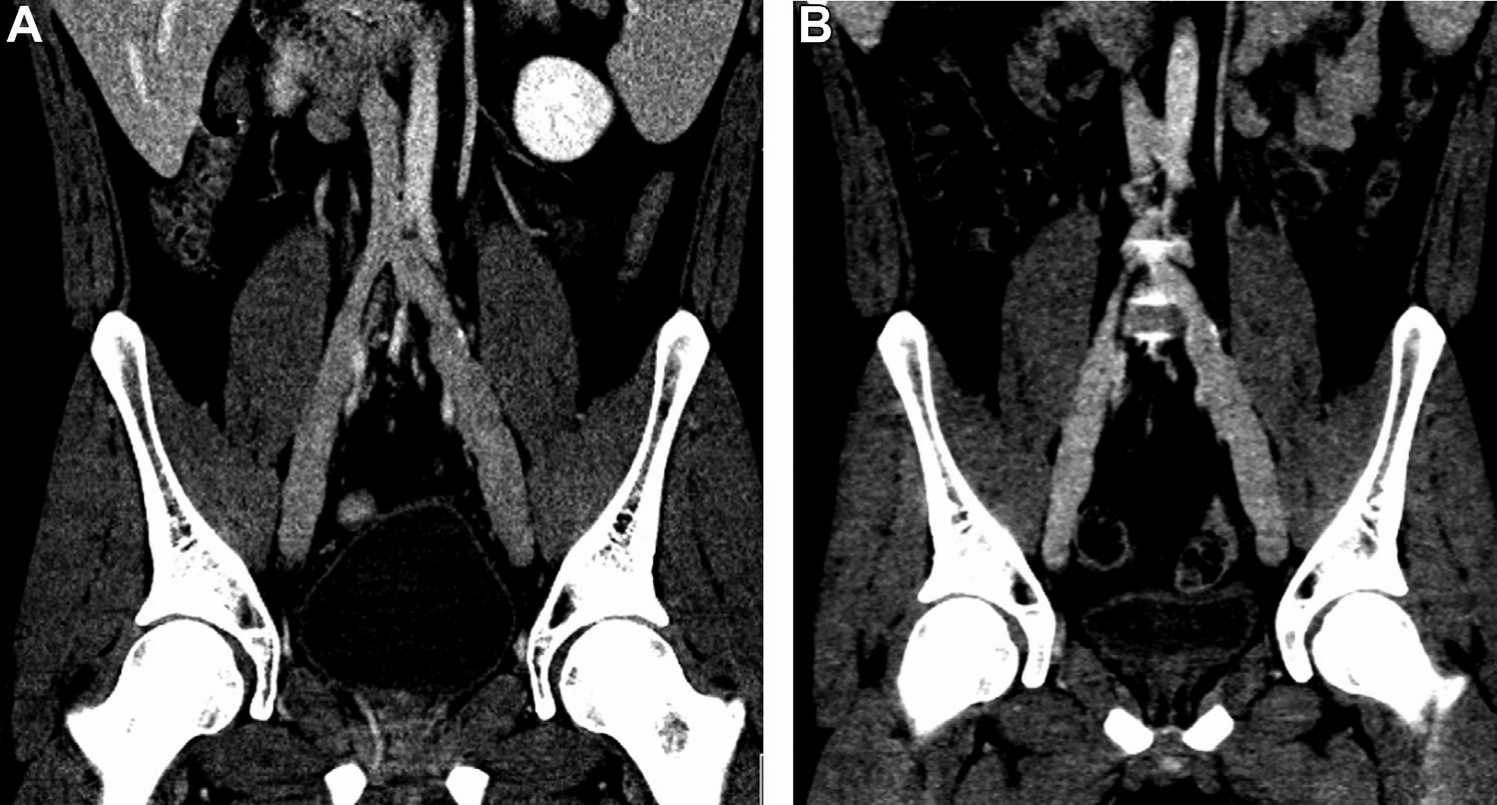
Postoperative images. **A,** Coronal computed tomography image showing bilateral external iliac veins 6 months after repair. **B,** Coronal computed tomography images showing bilateral external iliac veins 4 years after repair.

Computed tomography of the abdomen and pelvis was obtained 6 months postoperatively to guide anticoagulation management. The scan showed mild residual dilation bilaterally, as expected, with a maximal diameter of the external iliac vein of 1.9 cm on the right and 2.1 cm on the left without evidence of thrombosis (Fig 3, *A*). He was transitioned to aspirin, 81 mg daily, with annual duplex ultrasound. Computed tomography was performed 4 years postoperatively to allow for complete evaluation of the iliac venous system, which showed stable, mild residual ectasia (Fig 3, *B*). At last follow-up, he remained without symptoms.

## Discussion

Venous aneurysms are uncommon vascular abnormalities, with aneurysms of the iliac veins being particularly rare.^3^^,^^4^ Owing to their rarity, diagnosis and management has remained a challenge. These aneurysms are classified as primary when arising de novo or secondary when occurring due to trauma, arteriovenous fistula, or proximal venous outflow obstruction.^2^^,^^5^ Most iliac vein aneurysms are secondary aneurysms, most often occurring in the setting of an arteriovenous fistula.^1^

The only three previously reported patients with bilateral iliac vein aneurysms were very active athletes.^6^, ^7^, ^8^ Additionally, unilateral iliac vein aneurysms have been reported in patients who participated regularly in long-distance running and bicycling.^5^^,^^9^^,^^10^ Given the young age of our patient, bilateral involvement, and absence of iliac vein compression, we believe our patient had a congenital venous malformation. Rather than occurring as a direct result of our patient’s physical activity, the injury he had sustained from being an active adolescent had led to the incidental discovery of these asymptomatic aneurysms on the imaging study.

The presentation of these aneurysms is highly variable. In a recent review, 16% of patients were asymptomatic, with the aneurysms identified incidentally.^1^ Others have presented with unilateral extremity swelling, venous stasis, pain, abdominal mass or pain, testicular pain, back pain, and/or signs of venous insufficiency.^1^^,^^2^ However, the initial presentation of some patients will be secondary to the potentially fatal complications of these aneurysms, including pulmonary thromboembolism^10^, ^11^, ^12^ and rupture.^13^

Given the rarity of this condition, the approach to management has varied widely and has included observation, anticoagulation, and/or endovascular or open surgical intervention.^1^ Because of the known risks of these aneurysms and the young age of our patient, we elected for definitive surgical intervention. Although no approach has been standardized regarding the use of preoperative anticoagulation therapy, we administered preoperative anticoagulation therapy to decrease the risk of thrombosis while awaiting surgery, which also decreased the risk that intraoperative manipulation of the vein would lead to thromboembolism.

Multiple surgical techniques, including open and endovascular approaches, have been successfully used. The most commonly used technique for primary aneurysms has been tangential aneurysmectomy with lateral venorrhaphy.^8^^,^^13^, ^14^, ^15^, ^16^ Other approaches to primary aneurysms are shown in the Table. Given that bilateral repair was indicated for our patient, we performed primary repair to obviate the need for multiple prosthetic grafts or harvesting of multiple native veins. We used the stapler to resect a large portion of the aneurysm, followed by suture venoplasty, to effectively control the final size and shape of the vein. The repaired iliac veins were intentionally left slightly larger than normal to decrease the risk of thrombosis within a noncylindrical vein with intimal irregularity and imperfect laminar flow. In this setting, anticoagulation therapy was continued for 6 months postoperatively to further decrease the thrombotic risk. To the best of our knowledge, this approach has not been described for repair of an iliac vein aneurysm and certainly not for bilateral iliac vein aneurysms.

**Table:** Cases of primary iliac venous aneurysms reported in English literature

Investigator	Age, years; sex	Location	Presentation	Intervention	Follow-up period; outcome
Postma et al,^10^ 1989	33; M	L-IIV	Hemoptysis due to PE	Ligation	1 Year; asymptomatic
Petrunić et al,^14^ 1997	19; M	L-CIV	Extremity pain	Resection, venorrhaphy	1 Year; asymptomatic
Fourneau et al,^17^ 1998	21; F	L-CIV	Asymptomatic	Resection, reconstruction with femoral vein graft	18 Months; asymptomatic
Banno et al,^15^ 2004	20; F	L-EIV	Asymptomatic	Resection, venorrhaphy	16 Months; asymptomatic
Kotsis et al,^16^ 2008	31; F	L-EIV	Asymptomatic	Resection, venorrhaphy	24 Months; asymptomatic
Ysa et al,^2^ 2008	30; M	R-EIV	Pain and swelling of extremity	Anticoagulation	3 Months; mild edema
Humphries et al,^6^ 2010	32; F	B-EIV	Asymptomatic	None	20 Months; asymptomatic
Zou et al,^11^ 2011	14; F	L-EIV	Syncope due to PE	Anticoagulation	16 Months; asymptomatic
Jayaraj et al,^5^ 2012	37; F	L-EIV	Gluteal pain	Staple plication and aneurysm resection over a balloon mandrel	16 Weeks; asymptomatic
Hosaka et al,^12^ 2014	22; F	R-EIV	Dyspnea due to PE	Resection, patch venoplasty	8 Months; asymptomatic
Park et al,^13^ 2016	63; F	R-EIV	Shock due to rupture	Resection, venorrhaphy	52 Months; asymptomatic
Audu et al,^18^ 2017	63; M	L-IIV	Left testicular pain	Coil embolization	1 Month; asymptomatic
Yamamoto et al,^8^ 2019	50; M	B-EIV	Bilateral groin pain	Resection, venorrhaphy	8 Months; asymptomatic
van de Luijtgaarden et al,^19^ 2019	65; M	L-EIV	Swelling of extremity	Resection, tube graft replacement	No data
George et al,^20^ 2021	62; M	R-EIV	Asymptomatic	Observation	No data
Li et al,^21^ 2021	49; M	L-CIV	Back pain	Resection, venorrhaphy	No data

*B,* Bilateral; *CIV,* common iliac vein; *EIV,* external iliac vein; *F,* female; *IIV,* internal iliac vein; *L,* left; *M,* male; *PE,* pulmonary embolism; *R,* right.

## Conclusions

Iliac vein aneurysms are exceedingly rare but warrant strong consideration for intervention owing to the risk of potentially fatal complications. Staple aneurysmorrhaphy, combined with suture venoplasty, offers a safe and effective approach for the repair of iliac aneurysms, including bilateral aneurysms.
